# On-Premise Alcohol Establishments and Ambulance Calls for Trauma, Assault, and Intoxication

**DOI:** 10.1097/MD.0000000000003669

**Published:** 2016-05-13

**Authors:** Joel G. Ray, Linda Turner, Piotr Gozdyra, Flora I. Matheson, Burgess Robert, Emily Bartsch, Alison L. Park

**Affiliations:** From the Department of Medicine, St. Michael's Hospital, University of Toronto (JGR); Sunnybrook Centre for Prehospital Medicine, Sunnybrook Health Science Centre (LT, BR); Centre for Research on Inner City Health, St. Michael's Hospital (PG, FIM, ALP); and University of Toronto (EB), Toronto, Ontario.

## Abstract

Alcohol contributes to intentional and unintentional injury. We explored on-premise licensed alcohol establishments (LAEs) and emergency medical service (EMS) ambulance calls.

We completed a retrospective population-based study in the Region of Peel, Ontario, 2005 to 2014, where alcohol sales are tightly regulated and healthcare is universally available. We included participants age ≥ 19 years. Longitude–latitude coordinates of all 696 LAEs and all 267,477 EMS ambulance calls were ascertained, and then assigned to 1 of 1568 dissemination areas (DA) in Peel. Relative risks (RRs) described the association between density of on-premise LAEs (by DA deciles) and the rate of EMS calls, adjusted for material deprivation, and density of beer/liquor stores in each DA.

There was a curvilinear relation between LAE density and EMS calls for trauma, rising from 45.3 per 1000 in DAs with no LAEs to 381.0 per 1000 in decile-10 (adjusted RR 7.83, 95% confidence interval [CI] 6.15–9.97). This relation was more pronounced for alcohol-focused LAEs, and highest among younger males. Calls for assault (RR 2.67, 95% CI 1.26–5.65) and intoxication (RR 4.00, 95% CI 1.41–11.38) were more likely on the last day of the month and the day thereafter, compared to 1 week prior. At 02:00 hours, when LAEs must stop selling alcohol, there was a considerable rise in assault-related calls in DAs with LAE but not in DAs without LAEs.

On-premise LAEs contribute to EMS calls for trauma and assault, especially among young males, around last call, and when monthly pay cheques are cashed.

## Abstract 2

Supplemental Digital Content is available in the text

## BACKGROUND

Alcohol is a readily accessible and commonly consumed psychoactive substance^1^ that contributes worldwide to organ-specific disease, intentional and unintentional injury, and death.^2^ Alcohol impairs judgment and places a person at elevated risk of being both a victim and perpetrator of intentional injury.^3,4^ Many persons who overuse alcohol use the emergency department,^5^ and a large proportion of injury in the emergency department is attributable to hazardous alcohol use or recent alcohol ingestion.^6^ Additionally, emergency medical service (EMS) responders, including ambulance paramedics and firefighters, commonly deal with alcohol-related injury, both within the home and licensed alcohol establishments (LAEs). EMS superusers—those with at least 15 annual EMS encounters—are exponentially correlated with alcohol use and tend to be younger, male, and current tobacco users.^7^ Data show a strong association between alcohol intoxication and the risk of assault: those who are transported by EMS ambulance for alcohol intoxication are 9 times more likely to be transported for subsequent assault.^8^ Alcohol-related violence is most likely to occur between 22:00 and 02:00 hours,^9^ while others have observed a pronounced monthly cycle in alcohol-related hospital admissions^10^ and ambulance system calls^11^ related to some government transfer (welfare) programs.

The density of LAEs within communities—whether on-premise (e.g., bars and restaurants) or off-premise (e.g., liquor and beer stores)—is associated with intentional and unintentional injury.^3,12,13^ Citing the limitations of many prior studies on this topic, recommendations were recently published about ways to improve the data on alcohol sales outlets and adverse health outcomes.^14^ The list includes conducting analyses in areas under special license systems; stratifying outlets by categories (e.g., bars vs restaurants); incorporating highly localized clustering of outlets; and analyzing the shape of the relation between alcohol availability and outcomes, such as whether it is discrete or continuous, and linear versus curvilinear, to aid identification of target availability levels.^14^ Additionally, prior studies of on-premise alcohol outlets have not adjusted for the density of off-premise stores. Such adjustment is important because both on- and off-premise establishments are more likely to be found in densely populated areas and both can adversely influence health outcomes.

Herein, we determined the risk of EMS ambulance calls in association with the density of on-premise LAEs in the Region of Peel, Ontario, Canada, where all on-premise and off-premise outlets are licensed by the province of Ontario, and all EMS ambulance calls are under a single provider within a universal public healthcare system. In Ontario, the legal drinking age is strictly enforced at 19 years and older. We specifically evaluated EMS ambulance calls for intentional and intentional injury (henceforth, called “trauma”), including those due to assault, and categorized them by the type of alcohol LAE, the time of the EMS call, and the additive effect of tobacco sales at the same location as the LAE.^15^ We used deciles of on-premise LAE density, which offers better discrimination of target availability thresholds, thus addressing an important gap in the literature.^14^

### Study Design

We performed a retrospective population-based study of all EMS ambulance calls in the Region of Peel, Ontario, from January 1, 2005 through March 31, 2014. The Region of Peel contains about 1.4 million residents, https://en.wikipedia.org/wiki/Regional_Municipality_of_Peel. We used the 1617 dissemination areas (DA) in the Region of Peel as the geographic units for analysis. A DA is a standardized small, relatively stable geographic unit comprising a population of 400 to 700 persons, as determined by the Statistics Canada 2011 Census. We excluded 49 DAs with fewer than 100 residents per km^2^, to eliminate 1 major international airport and unpopulated areas with few or no LAEs or EMS ambulance calls, leaving 1568 DAs for analysis. Permission to complete the study was granted by the research ethics boards of St. Michael's Hospital and the Sunnybrook Health Sciences Centre.

### Exposure, Outcome Variables, and Covariates

The main exposure variable was the density of all on-premise LAEs, such as restaurants, bars, pubs, social clubs, and hotels. The list of on-premise LAEs, from 2005 to 2014, was provided by the Ontario Ministry of the Attorney General, as all are regulated under the provincial Liquor License Act, http://www.ontario.ca/laws/regulation/900719. The sale of alcohol at all LAEs must cease by 02:00 hours. Using address and postal code, the longitude and latitude coordinates of each LAE were determined. Each LAE was assigned to a given DA, using geocoding for Bing Maps, https://blogs.bing.com/maps/2013/12/17/how-to-fine-tune-location-coordinates-with-the-custom-geocoding-refinement-tool/. The density of LAEs was expressed as the number of LAEs per 1000 residents age ≥ 19 years per DA, and DAs were further assigned to deciles from the lowest density (decile 1 [D1]) to the highest (decile 10 [D10]). In most models, unless otherwise qualified below, each decile was compared to the reference group of DAs that had no LAEs (called “none”).

The main study outcome was the rate of EMS ambulance calls per 1000 residents age ≥ 19 years, separated by “trauma” and “medical” conditions, according to the EMS Problem field completed by each paramedic team on the Ambulance Call Report (ACR), http://www.ambulance-transition.com/pdf_documents/amb_call_report_completion_manual_v2.2_0603.pdf. EMS ambulance calls were obtained from the Peel EMS ACR database, housed at the Sunnybrook Centre for Prehospital Medicine. We excluded any EMS calls for patients age < 19 years, calls in which the pick-up address or location was not known, calls designated to transfer a patient between facilities, and calls in which no patient was found when EMS attended the scene, the patient was transported by another ambulance, or the call was cancelled before patient contact. Among trauma-related EMS ambulance calls, we further evaluated those classified on the ACR as an “assault,” namely, due to a physical assault, gunshot, or stabbing.

As a covariate, to describe DA-level poverty, we used the Material Deprivation Index quintile (Q),^16^http://www.torontohealthprofiles.ca/onmarg.php. The 2 other covariates were the density of off-premise alcohol outlets, namely, Beer Stores and Liquor Control Board of Ontario stores. Both comprise the majority of off-premise beer, wine, and spirit retail sales, and all are regulated under the Province of Ontario.^3,16^ In Ontario, alcohol cannot be sold in grocery or convenience stores.

We also obtained all licensed tobacco vendors from the Ontario Tobacco Inspection System database, which contains records on vendor compliance with tobacco sales requirement for all tobacco sales, including vending machines.^17^ Each vendor's address and postal code was used to assign it longitude and latitude coordinates.

### Data Analysis

An unadjusted relative risk (RR) and 95% confidence interval (CI) expressed the relation between density of on-premise LAEs (by DA deciles) and the rate of EMS calls, derived using negative binomial regression. The latter method is used for modeling over-dispersed count outcome variables, which was the case herein. The natural log of the number of residents age ≥ 19 years in each DA was used as the offset variable. DAs without any LAEs served as the referent. Adjusted RRs (aRR) were then calculated by adding material deprivation index quintile and density of off-premise outlets to all models.

We first explored the rate of EMS ambulance calls for trauma- and for medical-related conditions, in relation to the density of on-premise LAEs. We then examined the risk of trauma-related EMS ambulance calls in relation to the density of “alcohol-focused LAEs”—bars, taverns, nightclubs, and billiard/pool halls, which are expected to reflect greater alcohol consumption^18,19^—as well as in relation to the remaining LAEs, which are largely centered on food consumption and accommodation (Supplemental file S1). Any mixed bar–restaurant was classified as a restaurant. In the analysis of alcohol-focused LAEs and trauma-related EMS calls, we further explored the relation among males and females, by 10-year age groups.

We then restricted our dataset to all DAs with ≥1 LAE (i.e., D1–D10) and stratified by whether the EMS ambulance Pick-up Code was to a “bar or restaurant” versus “other.” Therein, we measured the aRR for the call being trauma-related or medical-related, as well as assault-related or not, with D1 serving as the referent in model. For each LAE in D1 to D10, we determined if there was a licensed tobacco vendor at the same spatial coordinates and calculated the aRR of medical-, trauma-, and assault-related EMS ambulance calls to LAEs with a tobacco vendor license versus LAEs without a tobacco vendor (the referent).

Many individuals in Ontario are paid near to the last day of the month, including those who receive social and disability assistance, http://www.durham.ca/departments/social/income_support/OWPaymentSchedule2014.pdf, in which about 82% to 85% is through direct deposit. Accordingly, for each LAE, we counted the number medical-, trauma-, assault-, and intoxication-related EMS calls on the last day of the month and the day thereafter (since LAEs serve until 02:00). We then compared those call-specific counts to the number of EMS ambulance calls at the same LAE, but 7 days earlier (i.e., on the 6th and 7th last days of the same month). Unadjusted RRs were generated using negative binomial regression with a repeated statement to account for the clustering of counts within each LAE.

Finally, we evaluated whether the rate of assault-related EMS ambulance calls differed by time of day. We plotted the proportion (95% CI) of assaults on a 24-h time clock, as a function of all EMS calls in that 24-h period. Plots were made for DAs with ≥1 LAE (i.e., D1–D10) and DAs without any LAEs. We repeated the above, but plotted the proportion of assaults as a function of all assault-related EMS ambulance calls in that 24-h period. For the time of day analyses, the timing of the EMS ambulance call was not recorded for 255 out of 3788 assault-related calls.

All statistical analyses were performed using SAS V.9.4 (SAS Institute Inc., Cary, NC).

## RESULTS

The study sample comprised 1568 DAs, containing 937,670 residents age ≥ 19 years (Table 1). There were 1332 (85%) DAs with no LAEs and 236 (15%) DAs with ≥1 LAE. For all 267,477 EMS ambulance calls, the respective mean ages of males and females were 57 and 58 years, but the respective ages were younger for trauma (51 and 57 years), intoxication (47 and 44 years), and especially, assault (36 and 37 years) (Table 1).

**TABLE 1:**
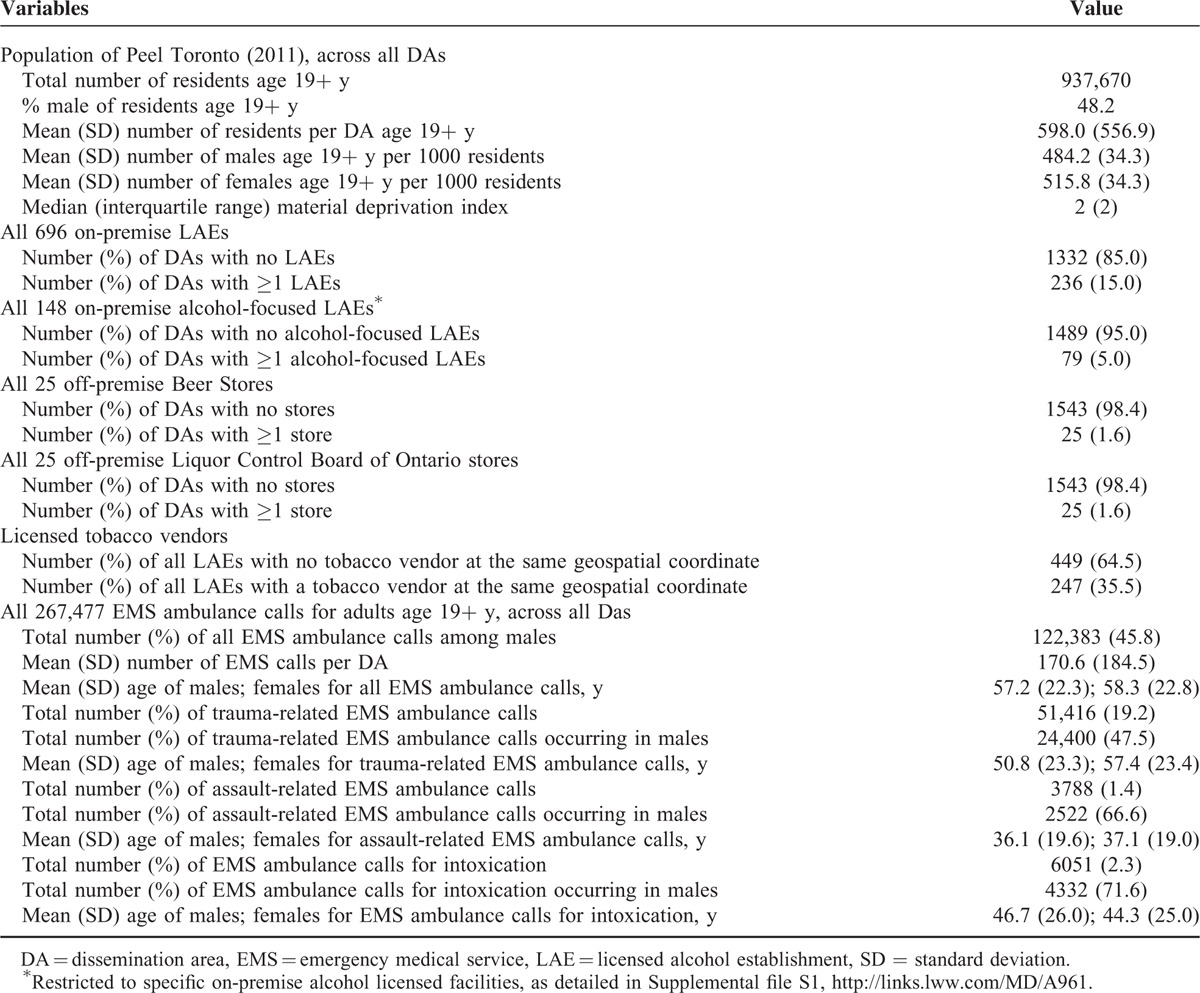
Description of the Included 1568 Dissemination Areas in the Region of Peel, Ontario, Canada, the On- and Off-Premise LAEs Therein, and the 267,477 Emergency Medical Service Ambulance Calls in the Region Over the Study Period

**FIGURE 1:**
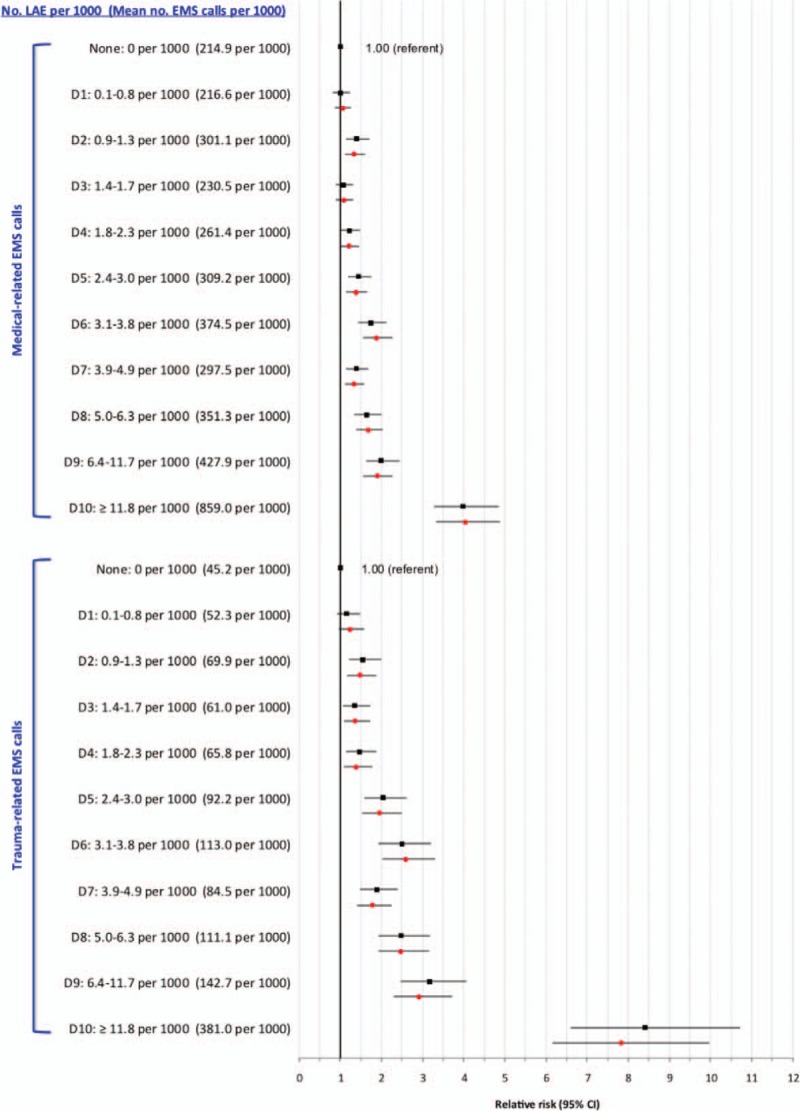
Rate and crude (black squares) and adjusted (red circles) relative risk for medical- and trauma-related emergency medical services (EMS) ambulance calls in relation to density of licensed alcohol establishments (LAEs). Shown are LAE by increasing density within dissemination areas, starting with areas without any LAE (the referent), and then by increasing deciles (D1–D10) of LAE density. Relative risks are adjusted for Material Deprivation Index quintile and density of off-premise licensed Beer Stores and Liquor Control Board of Ontario stores.

There was a curvilinear relation between LAE density and EMS ambulance calls, especially for trauma, rising from 45.3 per 1000 in DAs with no LAEs to 381.0 per 1000 in D10, a corresponding unadjusted RR of 8.41 (95% CI 6.60–10.72) and an aRR of 7.83 (95% CI 6.15–9.97) (Figure 1). Medical-related EMS ambulance calls followed the same pattern as for trauma, with higher absolute counts, but less pronounced RRs.

**FIGURE 2:**
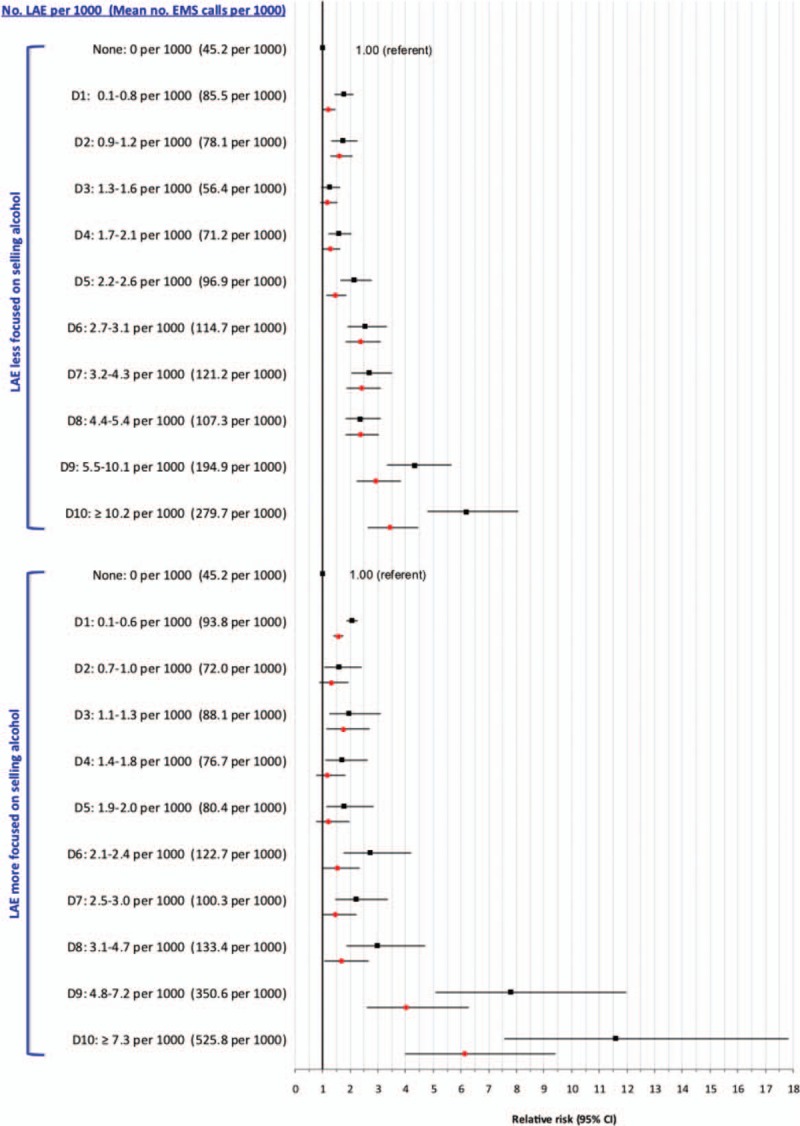
Rate and crude (black squares) and adjusted (red circles) relative risk for trauma-related emergency medical services (EMS) ambulance calls in relation to density of licensed alcohol establishments (LAEs) that are less and more focused on selling alcohol, as defined in Supplemental file 1. Shown are LAE by increasing density within dissemination areas, starting with areas without any LAE (the referent), and then by increasing deciles (D1–D10) of LAE density. Relative risks are adjusted for Material Deprivation Index quintile and density of off-premise licensed Beer Stores and Liquor Control Board of Ontario stores.

**FIGURE 3:**
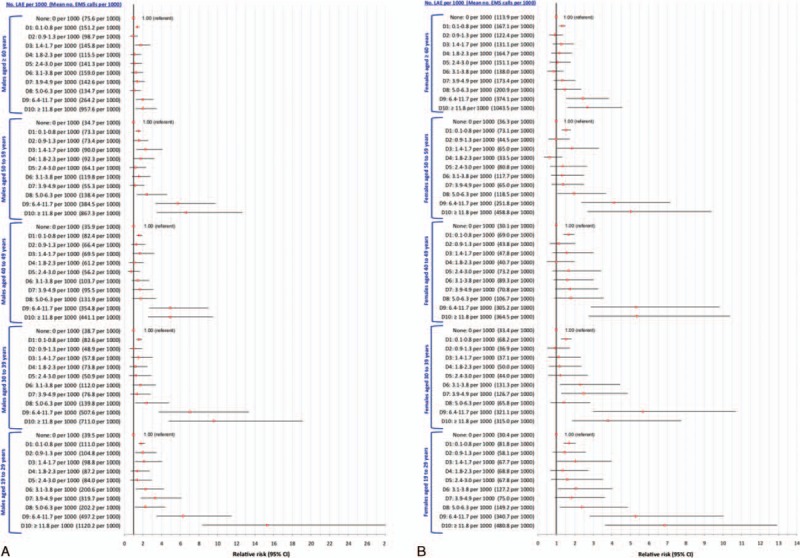
Rate and adjusted (black squares) and adjusted (red circles) relative risk for trauma-related emergency medical services (EMS) ambulance calls among males (A) and females (B) in relation to density of licensed alcohol establishments (LAEs) that are more focused on selling alcohol, as defined in Supplemental file 1. Shown are LAE by increasing density within dissemination areas, starting with areas without any LAE (the referent), and then by increasing deciles (D1–D10) of LAE density. Relative risks are adjusted for Material Deprivation Index quintile and density of off-premise licensed Beer Stores and Liquor Control Board of Ontario stores.

At D9 and D10 of LAE density, the absolute counts and aRR for trauma-related EMS ambulance calls were more pronounced for LAEs focused on selling alcohol than for other types of establishments (Figure 2). Upon restricting to alcohol-focused LAEs, the curvilinear association with trauma-related EMS ambulance calls was especially pronounced among younger males (Figure 3A) and females (Figure 3B). The rate of trauma-related EMS calls for males was much higher than for females. For example, among those age 19 to 29 years, the rate in D10 was 1120 per 1000 males, more than double the rate among females (480 per 1000).

**FIGURE 4:**
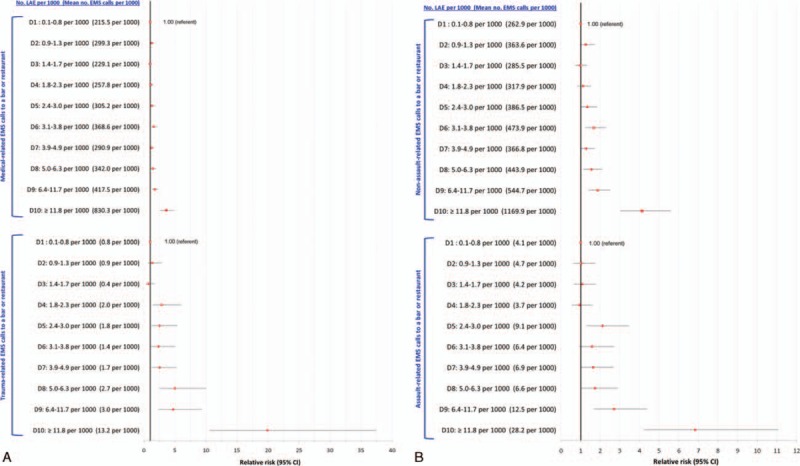
Rate and adjusted relative risk for emergency medical services (EMS) ambulance calls to a bar or restaurant that are trauma- or medical-related (A), as well as related or unrelated to assault (B). Shown are all licensed alcohol establishments (LAEs) within dissemination areas containing at least 1 LAE, and by increasing density of bars or restaurants within those dissemination areas, with decile 1 (D1) of density as the referent. Relative risks are adjusted for Material Deprivation Index quintile and density of off-premise licensed Beer Stores and Liquor Control Board of Ontario stores.

**FIGURE 5:**
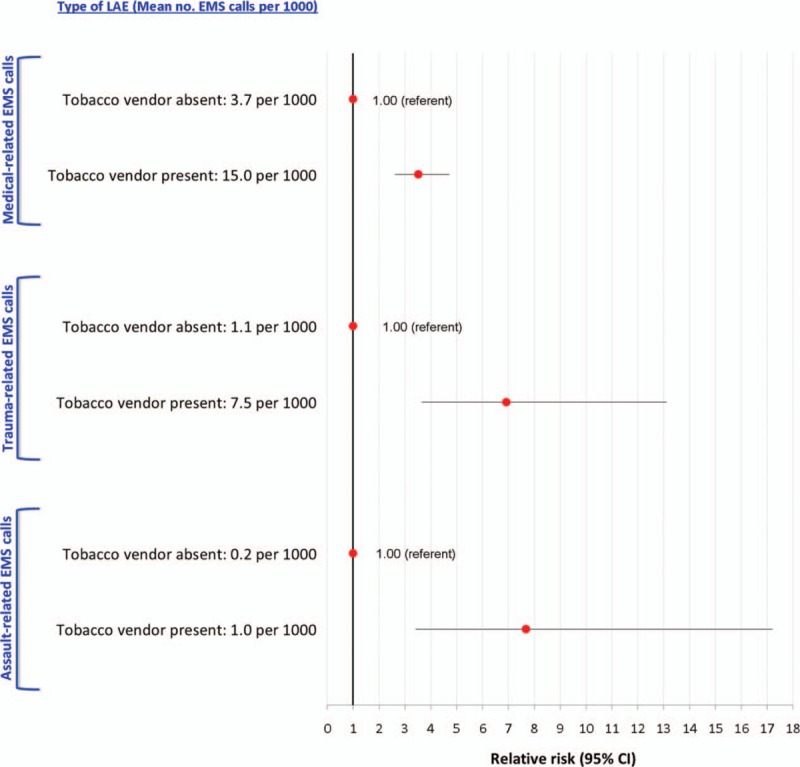
Rate and adjusted relative risk for medical-, trauma- and assault-related emergency medical services (EMS) ambulance calls in relation all licensed alcohol establishments (LAEs) with and without a licensed tobacco vendor at the same longitude and latitude coordinates. Shown are all LAEs within dissemination areas containing at least 1 LAE. Relative risks are adjusted for Material Deprivation Index quintile and density of off-premise licensed Beer Stores and Liquor Control Board of Ontario stores.

Upon restricting to EMS ambulance calls to a bar or restaurant within D1 to D10, the aRR was more pronounced for trauma-related than medical-related calls; albeit, medical-related calls were more common (Figure 4A). A similar pattern was seen for assault-related EMS ambulance calls (Figure 4B). Of all 696 LAEs within D1 to D10, 247 (35%) had a licensed tobacco vendor at the same spatial coordinates. The co-presence of a licensed tobacco vendor was associated with a 3.5 times higher risk of medical-related calls, and about a 7 to 8 times higher risk of trauma- and assault-related calls (Figure 5).

**FIGURE 6:**
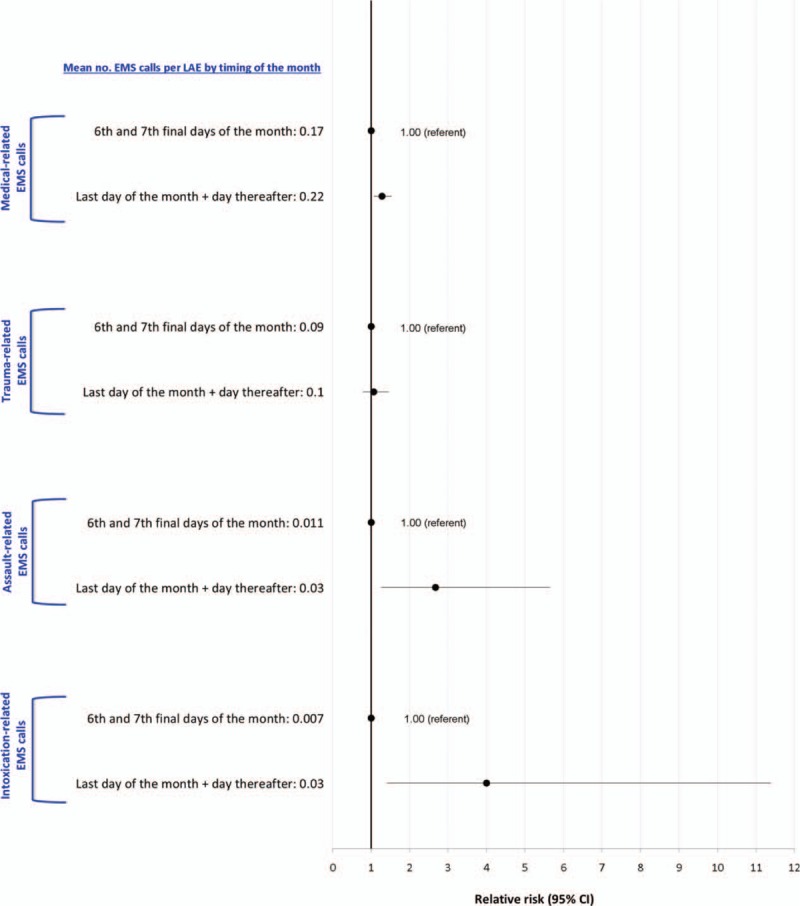
Mean number and unadjusted relative risk for medical-, trauma-, assault-, and intoxication-related emergency medical services (EMS) ambulance calls in relation to the last day of the month and the day thereafter, compared to 1 wk prior. Shown are all licensed alcohol establishments (LAEs) within dissemination areas containing at least 1 LAE.

**FIGURE 7:**
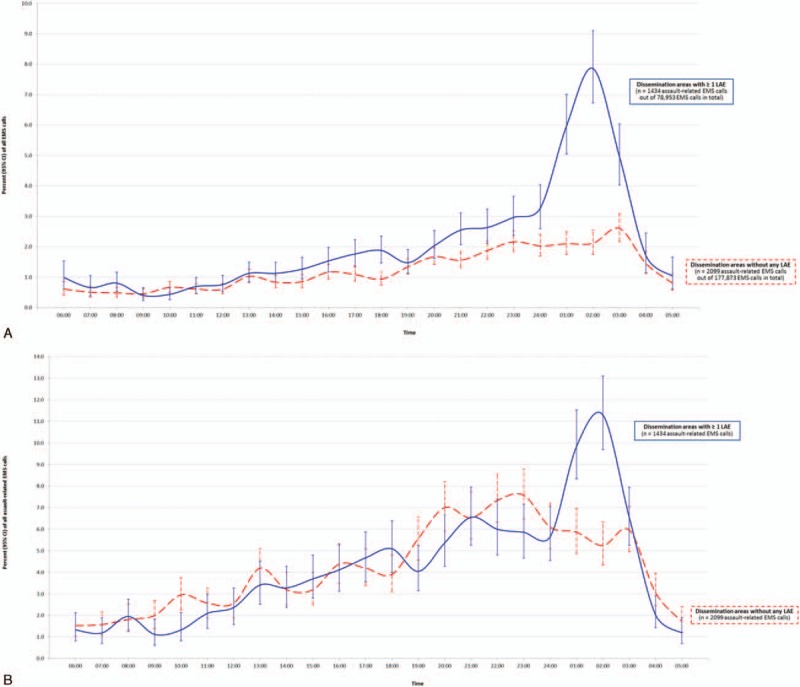
Assault-related EMS ambulance calls by time of day. Plotted is proportion (95% confidence interval) of assaults on a 24-h time clock, arising within dissemination areas (DA) with ≥1 licensed alcohol establishment (LAE) (blue solid line) or DA without any LAE (red dashed line). The proportion of assaults is presented as a function of all EMS calls (A), or of all assault-related EMS assaults (B). The timing of the EMS ambulance call was not reported for 255 out of 3788 assault-related calls. EMS = emergency medical services.

Intoxication-related EMS ambulance calls to an LAE were 4 times more likely on the last day of the month and the day thereafter, compared to 1 week prior; and the risk of assault was 2.7 times higher (Figure 6). A weaker association was observed for medical-related EMS ambulance calls, and none for trauma. Changing the time window to the last day of the month and the next 2 days thereafter, compared to the same 3 days in the week prior did not alter the findings (data not shown). The proportion of assault-related EMS ambulance calls increased gradually between 06:00 and 24:00 hours, but the proportion of EMS calls due to assault did not differ significantly different between DAs with (blue solid lines) and without (red dashed lines) on-premise LAEs (Figures 7A and B). However, by 02:00, the time at which the sale of all alcohol must stop, there was a considerable rise in assault-related calls in DAs with LAEs, but not in DAs without LAEs.

## DISCUSSION

In a setting with mandatory licensing of all LAEs and universal access to health care, including EMS services, we observed a 7.8 times higher risk of ambulance calls for trauma in areas with the highest density of on-premise LAEs, even after adjusting for retail off-premise outlets and a measure of area poverty. The RR for trauma was especially pronounced among young males in areas with a higher density of alcohol-focused LAEs, and for LAEs with a tobacco vendor at the same location. Calls for a victim of assault at a bar or restaurant were more pronounced—dramatically peaking at 02:00 hours—when the selling of alcohol must cease, and nearly tripled at the end of the month—when monthly pay cheques are usually deposited.

### Strengths and Limitations

An advantage of our study was the availability of a complete dataset of EMS ambulance calls within a universal public healthcare system, as well as all alcohol sales under a government-regulated system. Accordingly, we were able to respond to some of the recent recommendations about research on LAEs and adverse health outcomes.^14^ First, our study was solely conducted in an area under a single license system. Second, we stratified by the type of LAE. While we categorized a mixed bar–restaurant as a “restaurant,” doing so would attenuate the observed contrast seen between alcohol-focused LAEs and those less focused on alcohol. Third, we used highly specific geographic units—DAs—and we evaluated how the clustering of LAEs within DA deciles was related to EMS ambulance calls. The shape of the relation between LAE density and ambulance calls was curvilinear, with the highest risk largely confined to D9 and D10. We also adjusted for the density of beer and liquor retail outlets—both of which are also fully government regulated—to partly account for off-premise availability of alcohol.

Some of our findings may have been influenced by unmeasured ecological factors, such as the flow of vehicular and pedestrian traffic to and from a DA or LAE. For example, while individuals may gather inside and near LAEs located within busy DAs, they often do not live in those DAs. However, in our analysis of EMS ambulance calls by time of day, comparisons were between the same set of DAs, such that ecological factors would largely remain stable. In our analysis of EMS ambulance calls by time of month, each LAE served as its own control 7 days earlier (i.e., the same day of the week), thereby limiting confounding by spatial or temporal factors. In the latter analysis, the highest RR was for intoxication (Figure 7), strongly suggesting a temporary escalation in heavy alcohol use at the end of the month. In the same analysis, the RR was not significant for trauma-related EMS calls, which may be explained by the fact that trauma victims are more likely to be older and women, whereas intoxication and assault were more prevalent in younger men (Table 1). Another explanation is that alcohol consumption in older adults tends to occur at home rather than at an LAE.^20^ Yet, our dataset does not contain details about individual behavior or long-term alcohol use, something better evaluated using direct interviews and police reports,^21^ nor did we include patients under age 19 years or those who refused ambulance transport or left the scene before EMS arrival.

### Other Studies

The relation between alcohol availability—both on- and off-premise—and its negative health consequences is well understood.^2,22^ We showed that increasing deciles of LAE density was associated with a higher rate of all forms of EMS ambulance calls. This is in alignment with a systematic review of 44 studies on alcohol outlets density and 15 studies on the hours and days of operation of alcohol outlets, which showed that both are significantly associated with alcohol consumption, harmful drinking patterns, and injury.^23^ In a World Health Organization cross-sectional study of 5410 consecutive patients admitted to a hospital emergency department in 12 countries, 1 in 5 injury cases had alcohol involvement, 1 in 3 of latter cases were injured within 30 min of their last drink, and the injury was most often intentional, perpetrated by a stranger.^24^

The time of day in which alcohol-influenced injury arises appears to be near last call. In Ontario in 1996, legislation extended last call from 01:00 to 02:00, and there was a substantial rise in the risk of trauma cases unrelated to motor vehicle collisions.^25^ In our study, the peak in assaults was at 02:00, especially in DAs with ≥1 LAE, which is the time when an establishment must cease selling alcohol. In Norway, each 1-h extension of closing hours of on-premise alcohol outlets was associated with a 16% (95% CI 9–24) increase in police-reported assaults.^26^ Conversely, in Greater Newcastle, Australia, moving pub closing time from 05:00 to 03:30 resulted in fewer police-recorded assaults (RR 0.68, 95% CI 0.55–0.85).^27^ One explanation for the excess proportion of assaults at closing time may be the gathering of intoxicated patrons outside of LAEs. If proximate bars cater to different sorts of patrons, then this too may enhance the potential for confrontation outside of those establishments.^28^ Later closing hours enables some patrons to consume more alcohol, and it has been shown that hotels with later closing hours experience more assaults than those with standard business hours^29^; additional research is needed on the effects of later or staggered bar closing times.^19^

More than one-third of LAEs in our study had a tobacco vendor licensee at the location. It has been observed that residential areas with high levels of deprivation tend to have a greater density of outlets that sell alcohol and tobacco.^30^ However, we adjusted for Material Deprivation Index and found between a 3.5 to 7.7 times higher risk of EMS ambulance calls for medical, trauma, or assault in the co-presence of tobacco sales (Figure 5), something not been previously documented. There are biological and epidemiological reasons for why people co-misuse alcohol and nicotine.^31,32^ Bar patrons and smokers are more likely to be assaulted, since such individuals tend to take more risks.^19^ Hence, it is conceivable that the selling of tobacco serves as another marker of the so-called “bad bar,”^19^ in addition to our list of alcohol-focused LAEs (Figure 2 and Supplemental file S1).

### Policy Implications

While there are no randomized clinical trials to show that controlling the availability of alcohol leads to a reduction in intentional and unintentional injury, observational data are supportive of this notion.^2,24,33^ For example, the community of Buckhead, Atlanta petitioned its mayor and city council to enforce restrictions on the retail sales of alcohol, including the closure of many LAEs, while no such changes occurred in nearby Midtown or Downtown, Atlanta.^34^ With a 3.2% relative reduction in LAE density in Buckhead, there was an associated 2-fold greater reduction in violent crime compared to the control neighborhoods. Accordingly, though not causally proven, 2 means to achieving a reduction in EMS calls for injury and intoxication include limiting their hours of operation and their geographic density. Specifically, in areas with numerous LAEs, zoning by-laws might limit the issuing of further licenses. Using information such as presented in our study, 1 might focus on DAs with a high density of LAEs (e.g., those in D10), and apply such restrictions therein. Another approach might consider a risk-based liquor licensing framework, in which “bad bars”^19^—especially those with prior violations or offences related to the serving of alcohol—are subject to a cost-recovery element that includes higher fees for venues with higher risk.^35^ Some of these fees can help offset the higher costs for EMS calls.

Our data on timing of the month and EMS calls for assault or intoxication reinforce the observation that alcohol consumption is a function of not only its price, but also the availability of the funds to purchase alcohol.^11,36^ The risk of intoxication quadrupled at the end of the month, and the risk of assault nearly tripled. As confidently stated by others, and based on data from 112 studies, “[p]ublic policies that raise prices of alcohol are an effective means to reduce drinking.”^36^ It remains to be determined whether distributing payments over more than 1 time point in a month^10,11^ can reduce alcohol purchasing and alcohol-related harm. The latter could be tested within a cluster-based randomized clinical trial.

EMS personnel typically deal with serious injuries and medical conditions arising outside of hospital, some of which are related to alcohol consumption.^37^ As shown herein, 2.3% of EMS ambulance calls were for intoxication. One proposed strategy to better deal with the latter is the use of a checklist by EMS personnel, to triage intact individuals directly to a detoxification center. In a retrospective review of 718 EMS encounters, nearly 20% of intoxicated individuals were safely diverted to a detoxification center using this approach, with their checklist having a sensitivity of 99% (95% CI 97–100).^38^

A large proportion of EMS ambulance calls in our study were from trauma (19.4%), and specifically, assault (1.4%), and all victims were transported to an emergency department for further care. In such cases, when alcohol plays a role, there emerges an opportunity for a brief intervention to reduce future harm from alcohol consumption. For example, in 1 clinical trial, Crawford et al^39^ randomized patients received to receive an information leaflet versus the information leaflet plus an appointment with an alcohol health worker. At 12 months, the respective amount of alcohol consumed was 57.2 versus 70.8 units/wk.

## CONCLUSION

On-premise LAEs, especially those focused on the sale of alcohol, dramatically contribute to EMS ambulance calls for trauma and assault, especially among young males. Influential factors include the sale of tobacco products at the same location as the LAE, the 02:00 mandatory serving time limit of LAEs, and end-of month issuing of pay cheques.

## Supplementary Material

Supplemental Digital Content
