# Supplementary material for: On-Premise Alcohol Establishments and Ambulance Calls for Trauma, Assault, and Intoxication
Source: Medicine (Baltimore). 2016 May 13;95(19):e3669. doi: 10.1097/MD.0000000000003669 (PMC4902541; doi:10.1097/MD.0000000000003669)
Supplement: Supplemental Digital Content [file medi-95-e3669-s001.doc]

**Supplemental file S1. Type of on-premise licensed alcohol establishments. Those in bolded italics were considered herein to be “alcohol focused” licensed alcohol establishments.**

| Restaurant |
| --- |
| ***Bar/Sports Bar*** |
| Social Club |
| Hotel/Motel |
| ***Night Club*** |
| ***Bar /Tavern/Nightclub*** |
| Banquet Hall |
| Boat |
| ***Karaoke Bar/Restaurant*** |
| ***Billiard/Pool Hall*** |
| Live Theatre |
| Bowling Alley |
| Retirement Residence |
| ***Adult Entertainment*** |
| Educational Facility |
| ***Gaming Facility*** |
| ***Stadium*** |
| Golf Course |
| Motion Picture Theatre |
| Outdoor Area |
| Athletic Club |
| Military |
| Museum |
| Other |
| Railway Car |
